# Catarrhal Pneumonia

**Published:** 1887-05

**Authors:** G. W. Sherbino

**Affiliations:** Abilene, Texas


### CATARRHAL PNEUMONIA.

G. W. Sherbino, M. D., Abilene, Texas.

Case I.—Cora G., æt. three. Was called at nine A. M. Found child with pulse 180, temperature 103.3°, respirations 60 per minute. Starting jumping when falling asleep; face flushed; throbbing of vessels in the neck. ℞. Bell.$^{4m}$, one dose. S. L. every hour.

Third day.—Passed a very restless night; coughing, causing, or seeming to cause, pain in the chest; groaning with every breath; cries whenever being moved. ℞. Bry. A.$^{1m}$, one dose.

Fourth day.—Pulse 165, temperature 103°, respiration 60. Cross when waking, kicks and acts naughty. Red sand in the urine. Four to eight, exacerbation. "*Fan-like motion* of *alæ nasi* (Baptisia Phos.). ℞. Lycopod, ($^{1400}$).

Fifth day.—Pulse 150, respiration 60. Somewhat better today, mentally.

Sixth day.—Convalescent.

On the seventh day she was exposed to cold draught. In the evening all the symptoms came back again. ℞. Lycop.$^{30m}$, one dose, cured.

CASE II.—Flora P., æt. four. I was called at nine A. M. to see a sick little girl who had had a cough for a few days, but the night before she had a high fever, and I found her in the same condition. Pulse 180, temperature 104°, respiration 40. She was stupid, and yet she would start and jump. Mother was afraid of spasms. (℞. (Bell.$^{4m}$), one dose.

Second day.—Not quite so nervous. Did not start and jump so much. Temperature, 104½, pulse 160, respiration 60 per minute. Hacking cough all the time.

I noticed *now fan-like motion* of *the alæ nasi.* We looked at the urine and found the red sand in the bottom, and she had to-day the four to eight exacerbation. ℞. Lycopod. ($^{1400}$), one dose.

Third day.—The same.

Fourth day.—No change. ℞. One dose of Lycopd.$^{30000}$. S. L. every half hour. At eleven A. M. the first dose was given of the (30 M). At four P. M. I was sent for; child worse. I saw an exacerbation. Father wants to call an allopath. I told them he might help me on diagnosis, but we could not agree on the treatment. I returned at nine P. M. Found the regular there. The temperature at four P. M. was 104½°, pulse 160, respiration 60. But now, at nine P. M., temperature 102, respiration 50, pulse 140. Child brighter every way. The allopath says she is getting better, and will be well in a few days.

She was convalescent on the sixth day.

Conclusion:

1st. The 1400 did not cause any exacerbation, but the 30,000 did.

2d. If I had repeated the remedy every two hours (secundum artem), I would have kept up the aggravation, and the family would have been unnecessarily alarmed, and the doctor called would have carried off the laurels.

3d. One dose saved my reputation in the family and in the neighborhood.

4th. Give the drug time to act, and exhaust its action before repeating.

5th. Give the simillimum in the minimum dose, and relief both prompt and sure will come.

---

In the above article Dr. Sherbino gives Baptisia, Lyc., Phos. as having fan-like motion of wings of nose. To these we add Bromine and Ant. tart. See Hering's *Guiding Symptoms.*—EDS.

